# Molecular recircumscription of *Broussonetia* (Moraceae) and the identity and taxonomic status of *B. kaempferi* var. *australis*

**DOI:** 10.1186/s40529-017-0165-y

**Published:** 2017-02-21

**Authors:** Kuo-Fang Chung, Wen-Hsi Kuo, Yi-Hsuan Hsu, Yi-Hsuan Li, Rosario Rivera Rubite, Wei-Bin Xu

**Affiliations:** 1 Research Museum and Herbarium (HAST), Biodiversity Research Center, Academia Sinica, 128 Academia Road, Section 2, Nangang, Taipei, 11529 Taiwan; 2 School of Forestry and Resource Conservation, National Taiwan University, No. 1, Sec. 4, Roosevelt Rd., Taipei, 10617 Taiwan; 3 Department of Biology, College of Arts and Sciences, University of the Philippines Manila, Padre Faura, 1000 Manila, Philippines; 4 Guangxi Key Laboratory of Plant Conservation and Restoration Ecology in Karst Terrain, Guangxi Institute of Botany, Guangxi Zhuang Autonomous Region and Chinese Academy of Sciences, Guilin, 541006 China

**Keywords:** *Allaeanthus*, *Broussonetia* ×*kazinoki*, *Broussonetia monoica*, Dorstenieae, Lectotype, Neotype, Paper mulberry genus, Taxonomy

## Abstract

**Background:**

Despite being a relatively small genus, the taxonomy of the paper mulberry genus *Broussonetia* remains problematic. Much of the controversy is related to the identity and taxonomic status of *Broussonetia kaempferi* var. *australis*, a name treated as a synonym in the floras of Taiwan and yet accepted in the floras of China. At the generic level, the monophyly of Corner (Gard Bull Singap 19:187–252, 1962)’s concept of *Broussonetia* has not been tested. In recent studies of *Broussonetia* of Japan, lectotypes of the genus were designated and three species (*B. kaempferi*, *Broussonetia monoica*, and *Broussonetia papyrifera*) and a hybrid (*B.* ×*kazinoki*) were recognized. Based on the revision and molecular phylogenetic analyses, this article aims to clarify these issues.

**Results:**

Herbarium studies, field work, and molecular phylogenetic analyses indicate that all Taiwanese materials identifiable to *B. kaempferi* var. *australis* are conspecific with *B. monoica* of Japan and China. Molecular phylogenetic analyses showed that *Broussonetia* sensu Corner (Gard Bull Singap 19:187–252, 1962) contains two clades corresponding to sect. *Broussonetia* and sect. *Allaeanthus*, with *Malaisia scandens* sister to sect. *Broussonetia*.

**Conclusions:**

Based on our analyses, *B. kaempferi* var. *australis* is treated as a synonym of *B. monoica* and that *B. kaempferi* is not distributed in Taiwan. To correct the non-monophyly of *Broussonetia* sensu Corner (Gard Bull Singap 19:187–252, 1962), *Broussonetia* is recircumscribed to contain only sect. *Broussonetia* and the generic status of *Allaeanthus* is reinstated.

**Electronic supplementary material:**

The online version of this article (doi:10.1186/s40529-017-0165-y) contains supplementary material, which is available to authorized users.

## Background

Prior to Corner (1962)’s circumscription, *Broussonetia* L’Hér. ex Vent. was known as a genus of three species distributed in East Asia and continental Southeast Asia: the type species *Broussonetia papyrifera* (L.) L’Hér. ex Vent., *Broussonetia kaempferi* Siebold, and *Broussonetia kazinoki* Siebold (Ohwi 1965; Liu and Liao 1976), with a hybrid between *B. kazinoki* and *B. papyrifera* known from Japan (Kitamura and Murata 1980; Yamazaki 1989; Okamoto 2006) and Korea (Yun and Kim 2009). Corner (1962) expanded the generic concept by combining *Allaeanthus* Thwaites as *Broussonetia* sect. *Allaeanthus* (Thwaites) Corner, stating that “*there are no major differences between these sections* (i.e., sect. *Broussonetia* and sect. *Allaeanthus*), *which are not generically distinct*” (Corner 1962). Currently, *Broussonetia* sect. *Allaeanthus* comprises four species: *B. greveana* (Baill.) C.C. Berg of Madagascar, *B. kurzii* (Hook. f.) Corner of China (Yunnan), India (Assam), Myanmar, and Thailand, *B. luzonica* (Blanco) Bureau of the Philippines and Sulawesi, and *B. zeylanica* (Thwaites) Corner of Sri Lanka (Corner 1962; Berg 1977; Zhou and Gilbert 2003; Berg et al. 2006). Based on Corner (1962)’s circumscription, *Broussonetia* is characterized by membranous stipules, globose syncarps, drupes covered by thickly sets of slender stalked bracts of various shapes, crustaceous to ligneous endocarps, and conduplicate to plane cotyledons. Although Corner (1962)’s expanded concept has been followed by most authors (e.g., Berg 1977; Rohwer 1993; Chang et al. 1998; Zhou and Gilbert 2003; Berg et al. 2006) except for Capuron (1972) who sustained the generic status of *Allaeanthus*, the monophyly of *Broussonetia* sensu Corner (1962) has not yet been tested (Zerega et al. 2005; Clement and Weiblen 2009) and much about the taxonomy of the genus remains unsettled.

Commonly known as paper mulberry, *Broussonetia papyrifera* is renowned as a fibrous tree essential to the development of paper making technique in ancient China around 100 A.D. (Ling 1961; Barker 2002). Long before Linnaeus’ time, paper mulberry had been cultivated widely in European gardens (Barker 2002) and, as documented during Captain James Cook’s circum-Pacific voyages, clonally propagated across Remote Oceanic islands by Austronesian-speaking peoples for making bark cloth (*tapa*), a non-woven textile that is highly symbolic of Austronesian material culture (Matthews 1996; Whistler and Elevitch 2006; Seelenfreund et al. 2010). This fast-growing dioecious weedy tree species is most likely native to China, Taiwan, and continental Southeast Asia (Matthews 1996); however, because of its long history of utilization (Matthews 1996; Barker 2002; Chang et al. 2015), considerable discrepancies exist in the literature regarding distribution ranges of *B. papyrifera* (Table 1). Based on the phylogeographic analysis of chloroplast *ndhF*-*rpl32* intergenic spacer, Chang et al. (2015) demonstrated that Pacific paper mulberry originated in southern Taiwan, providing the first ethnobotanical support for the “out of Taiwan” hypothesis of Austronesian expansion. Peñailillo et al. (2016) further showed that Pacific paper mulberries are predominately female, consolidating reports on the clonal nature and corroborating Chang et al. (2015)’s inference. In addition to its long-fiber, this fast growing weedy tree has also been introduced for erosion control worldwide (Matthews 1996). Consequently, the multipurpose paper mulberry has been naturalized in southern Europe and become invasive in Argentina, Ghana, Uganda, Pakistan, the Philippines, Solomon Islands, and USA. (Matthews 1996; Barker 2002; Morgan and Overholt 2004; Florece and Coladilla 2006; Whistler and Elevitch 2006; Marwat et al. 2010; Bosu et al. 2013).

**Table 1:** Distribution of *Broussonetia papyrifera* in selected literatures

Kanehira (1936)	Taiwan, Myanmar, Thailand, Malaysia, Pacific islands, China, Japan
Chûjô (1950)	Japan, Korea, China, Ryukyus, Taiwan, Philippines, Vietnam, Thailand, Myanmar, India, Malay, Sumatra, Java, Borneo, SW Pacific islands, Europe, North America, Australia
Liu (1962)	Taiwan, India, Thailand, Malaysia, Pacific islands, Japan, China
Li (1963)	Taiwan, Indo-Malaysia, China, Japan to the Pacific islands, Taiwan
Ohwi (1965)	Cultivated for making paper in Japan (Honshu, Shikoku, Kyushu); Ryukyus, Formosa, China, Malaysia
Liu and Liao (1976)	China, Japan, the Pacific Islands, Malaysia, Thailand and India
Kitamura and Murata (1980)	Central and southern China, Taiwan, Vietnam, Thailand, Myanmar, India, Malaysia, Pacific Islands
Yamazaki (1982)	S. China, Taiwan, Indochina, Thailand, Burma and Malaysia. Cultivated in Japan
Yamazaki (1989)	Central and southern China, Indochina, Malaysia
Liao (1991, 1996)	Taiwan, Southern China, Japan, the Pacific Islands, Indochina, Malaysia, Thailand, Burma and India
Liu et al. (1994), Lu et al. (2006)	Central and southern China, Taiwan, Japan, Malay, Pacific islands
Matthews (1996)	Japan, Korea, northern, central, and southern China, Taiwan, Vietnam, Laos, Thailand, Cambodia, Myanmar, India (Sikkim), islands Southeast Asia (excluding the Philippines and Borneo), Melanesia, and Polynesia islands
Florence (1997)	Native to China and Japan, widely cultivated in South East Asia, Malaysia and the Pacific
Shimabuku (1997)	Cultivated and escaped in Ryukyus. China, Taiwan, Indochina, Malaysia
Chang et al. (1998)	Distributed throughout China from the north to south, also in Sikkim, Myanmar, Thailand, Vietnam, Malaysia, Japan, Korea, wild or cultivated
Cao (2000)	China (Gansu, Shananxi, Shanxi, Henan, Hebei, Shandong, Jiangsu, Anhui, Zhejiang, Fujian, Jiangxi, Hubei, Hunan, Guangdong, Hainan, Guangxi, Guizhou, Yunnan, Sichuan, Xizang), Taiwan
Barker (2002)	East Asia, in China, Japan, and Korea
Zhou and Gilbert (2003)	China, Taiwan, Cambodia, Japan, Korea, Laos, Malaysia, Myanmar, Sikkim, Thailand, Vietnam; Pacific Islands
Berg et al. (2006)	India (Assam), China (incl. Taiwan), Indochina, Japan (introduced in the Ryukyu Islands), Myanmar, Thailand, Polynesia; in Malesia: introduced in Sumatra, Java, Philippines, Celebes, Lesser Sunda Islands (Flores, Timor, Alor, Wetar), Moluccas, New Guinea
Okamoto (2006)	Japan (cultivated and naturalized), Taiwan, S. China, Indochina, India, the Malesian region and Pacific islands
Whistler and Elevitch (2006)	Native to Japan and Taiwan; an ancient introduction to many Pacific islands as far east as Hawai‘i
Yun and Kim (2009)	Korea, Japan, China, Taiwan, Malaysia, Laos, Myanmar, Thailand, Vietnam
LaFrankie (2010)	China, Japan, naturally occurring as far south as Myanmar and Thailand, cultivated in Java, not found either in Malay or Borneo

Although paper mulberry has long been introduced to Europe (Barker 2002), it is Kaempfer (1712)’s plate (“*Kampf. amoen.* 471. *t.* 472”) depicting paper mulberry (as “*Morus papyrifera*”) in Japan cited by Linnaeus (1753) that was lectotypified (Florence 1997) for *Morus papyrifera* L., the basionym of *Broussonetia papyrifera*. In Japan where paper mulberry is known as “Kajino-ki” (Okamoto 2006), *B. papyrifera* has long been regarded as non-native (Schneider 1917), also introduced for paper making around ca. 610 A.D. (Matthews 1996; Barker 2002). Quite confusingly, the name Kajino-ki was taken by Siebold (1830) for *B. kazinoki*, a name long applied to a small ‘monoecious’ shrub with ‘globose’ staminate catkins ca. 1 cm across known as Hime-kôzo in Japan (Chûjô 1950; Kitamura and Murata 1980; Yamazaki 1989; Okamoto 2006). Elsewhere, *B. kazinoki* is also widely found in China (Chang et al. 1998; Zhou and Gilbert 2003), Taiwan (Liao 1989, 1991, 1996), and Korea (Yun and Kim 2009). The natural hybrid between Hime-kôzo and Kajino-ki known as Kôzo in Japan (as *B. kazinoki* × *B. papyrifera*; Kitamura and Murata 1980; Okamoto 2006) and Daknamu in Korea (Yun and Kim 2009) has also been long cultivated and favored by Japanese and Korean farmers for traditional paper making for centuries (Yamazaki 1989). In 2009, this natural hybrid was further named *B.* ×*hanjiana* M. Kim (Yun and Kim 2009). The third species, *B. kaempferi*, is a ‘dioecious’ lianascent climber with ‘spicate’ staminate catkins ca. 1.5–2.5 cm long distributed in Japan (known as Tsuru-kôzo), central to southern China, and Vietnam (Ohwi 1965; Yamazaki 1982; Zhou and Gilbert 2003; Okamoto 2006), with a controversial record in Taiwan (Suzuki 1934; Kanehira 1936; Liu and Liao 1976; Liao 1989, 1991, 1996).

In the article titled ‘A speciograhical revision on *Broussonetia kazinoki*’, Suzuki (1934) studied a set of highly variable specimens akin to “Hime-kôzo” collected from Taiwan first identified as *B. kaempferi* sensu Forbes and Hemsley (1894) by Hayata (1911). After comparing with specimens collected from Japan, Suzuki (1934) concluded that *B. kazinoki* and *B. kaempferi* are different species and that all the Taiwanese specimens should be collectively recognized as a distinct taxon, which he named *B. kaempferi* var. *australis* T. Suzuki. However, Suzuki (1934)’s treatment was not cited in Kanehira (1936), the most influential pre-World War II work on the woody flora of Taiwan (Li 1963). Instead, Kanehira (1936) followed Hayata (1911)’s treatment, identifying the entity as *B. kaempferi* and stating that the species is dioecious. Interestingly, although a majority of the treatments of Kanehira (1936)’s ‘Formosan Trees’ were followed in the first edition of the Flora of Taiwan (Liu and Liao 1976) and its predecessor (Liu 1962), both Liu (1962) and Liu and Liao (1976) treated the species as *B. kazinoki,* with *B. kaempferi* var. *australis* synonymized under *B. kazinoki* [though mistakenly typed as *B.* “*kazinoki*” Sieb. var. *australis* Suzuki in Liu and Liao (1976)]. Subsequently, Yamazaki (1982) revisited the issue. Yamazaki (1982) emphasized the differences in leaf shapes, adopting Suzuki (1934)’s treatment by circumscribing *B. kaempferi* var. *kaempferi* as a variety endemic to Japan and *B. kaempferi* var. *australis* a variety distributed in southern China, Taiwan, and Vietnam. Yamazaki (1982)’s treatment was adopted by most treatments of the Chinese floras (e.g., Chang et al. 1998; Zhou and Gilbert 2003; Liu and Cao 2016) with rare exceptions such as Cao (2000) in which *B. kaempferi* var. *australis* was treated as a synonym of *B. kaempferi*. The taxonomic status of *B. kaempferi* var. *australis* was further complicated when Liao (1989, 1991, 1996), in addition to *B. kazinoki*, reported *B. kaempferi* from Taiwan, with *B. kaempferi* var. *australis* again treated as a synonym of *B. kazinoki*. Liao (1989, 1991, 1996)’s treatment has been followed by all subsequent works of Taiwan (Liu et al. 1994; Yang et al. 1997; Lu et al. 2006) as well as local online blogs (e.g., Nature Campus http://nc.kl.edu.tw/bbs/index.php). In a recent assessment of the conservation status of the flora of Taiwan, *B, kaempferi* is listed as a ‘vulnerable’ species with its small and declining populations (Wang et al. 2015).

Given the complicated taxonomy of these names, it is rather surprising that none of the abovementioned authors had attempted to examine and clarify type materials of the two names described by Siebold (1830) as well as *B. kaempferi* var. *australis*. After lectotypifying Siebold’s Japanese plant names (Akiyama et al. 2013), Ohba and Akiyama (2014) revised the taxonomy of *Broussonetia* of Japan. Surprisingly, the specimen of Siebold’s collections of Japanese plants that matched best to the protologue of *B. kazinoki* and thus lectotypified (M-0120984) turned out to be Kôzo (Akiyama et al. 2013; Ohba and Akiyama 2014), the natural hybrid between Hime-kôzo and Kajino-ki cultivated for traditional paper making. Consequently, *B. monoica* Hance, the next valid name long synonymized under *B. kazinoki* (e.g., Zhou and Gilbert 2003) becomes the correct name for Hime-kôzo (Ohba and Akiyama 2014). For *B. kaempferi*, the plate of ‘*Papyrus spuria*’ in Kaempfer (1712) was lectotypified (Akiyama et al. 2013). Based Ohba and Akiyama (2014)’s treatment, the four species of *Broussonetia* in Japan are *B. kaempferi* (Tsuru-kôzo), *B.* ×*kazinoki* (Kôzo), *B. monoica* (Hime-kôzo), and *B. papyrifera* (Kajino-ki).

Because Ohba and Akiyama (2014) dealt only with Japanese materials, this study attempts to clarify the distribution range of *B. papyrifera* and resolve controversies surrounding the name *B. kaempferi* var. *australis* based on herbarium work, field observation, and molecular data. We also sampled species of *Broussonetia* sect. *Allaeanthus* which thus far has never been sampled (e.g., Zerega et al. 2005; Clement and Weiblen 2009) to test the monophyly of *Broussonetia* sensu Corner (1962).

## Methods

### Taxon sampling

Herbarium specimens of A, BM, E, GH, HAST, K, TAI, TAIF, and TNM (herbarium acronyms according to Index Herbariorum; Thiers 2016) were examined. Specimen images of Naturalis Biodiversity Center (http://bioportal.naturalis.nl/?language=en&back), the Chinese Virtual Herbaria (http://www.cvh.org.cn/), and Global Plants on JSTOR (http://plants.jstor.org/) were consulted. Fieldtrips were conducted in Taiwan, China (Zhejiang, Fujian, Guangdong, and Guangxi), and the Philippines. All voucher specimens were deposited in HAST. To expand geographic range of our taxon sampling, herbarium collections were also sampled with the permission from E, HAST, Harvard University Herbaria (A and GH), TAIF, and TNM. The HTTP URIs of the images of important (types and vouchers) specimens examined are listed in Table 2.

**Table 2:** HTTP URIs of specimens examined (e.g., Hyam et al. 2012)

Species	Collector name and no. (Herbarium barcode)	HTTP URI	Type status	Current identification
*B. kaempferi* var. *australis*	*T. Suzuki 8362* (TAI-118781)	http://tai2.ntu.edu.tw/Specimen/specimen.php?taiid=118781	Holotype?	*B. monoica*
*B. kaempferi* var. *australis*	*S. Suzuki 6042* (TAI-037623)	http://tai2.ntu.edu.tw/Specimen/specimen.php?taiid=037623	Paratype	*B. monoica*
*B. kaempferi* var. *australis*	*T. Suzuki 8952* (TAI-037637)	http://tai2.ntu.edu.tw/Specimen/specimen.php?taiid=037637	Paratype	*B. monoica*
*B. kaempferi* var. *australis*	*T. Suzuki 4629* (TAI-037629)	http://tai2.ntu.edu.tw/Specimen/specimen.php?taiid=037629	Paratype	*B. monoica*
*B. kaempferi* var. *australis*	*T. Suzuki 10827* (TAI-037634)	http://tai2.ntu.edu.tw/Specimen/specimen.php?taiid=037634	Paratype	*B. monoica*
*B. kaempferi* var. *australis*	*S. Suzuki 5998* (TAI-037627)	http://tai2.ntu.edu.tw/Specimen/specimen.php?taiid=037627	Paratype	*B. monoica*
*B. kaempferi* var. *australis*	*T. Suzuki 6841* (TAI-037638)	http://tai2.ntu.edu.tw/Specimen/specimen.php?taiid=037638	Paratype	*B. monoica*
*B. kaempferi* var. *australis*	*S. Suzuki 3848* (TAI-037630)	http://tai2.ntu.edu.tw/Specimen/specimen.php?taiid=037630	Paratype	*B. monoica*
*B. kaempferi*	*T. Tanaka & Y. Shimada 13557* (PH-00065996)	http://tai2.ntu.edu.tw/specimen/specimen.php?taiid=65996	Voucher cited in Liao (1989,1991, 1996)	*B. monoica*
*B. kaempferi*	*T. Tanaka & Y. Shimada 13557* (PH-00065997)	http://tai2.ntu.edu.tw/specimen/specimen.php?taiid=65997	Voucher cited in Liao (1989, 1991, 1996)	*B. monoica*
*B. kaempferi*	*Y. Yamamoto s.n.* 1929 (TAI-037610)	http://tai2.ntu.edu.tw/specimen/specimen.php?taiid=037610	Voucher cited in Liao (1989, 1991, 1996)	*B. monoica*
*B. monoica*	*B. C. Henry 21933* (BM-000895739)	http://plants.jstor.org/stable/10.5555/al.ap.specimen.bm000895739	Holotype	*B. monoica*
*B. kazinoki*	*P. F. von Siebold s.n.* 1842 (M-0120984)	http://plants.jstor.org/stable/10.5555/al.ap.specimen.m0120984	Lectotype	*B.* ×*kazinoki*
*Ampalis greveanus* Baill.	*Grevé 254* (P-00108324)	http://mediaphoto.mnhn.fr/media/1441450681482QFbvIibvcIYZxWVk	Lectotype	*Allaeanthus greveanus*
*Ampalis greveanus* Baill.	*Grevé 254* (P-00108325)	http://mediaphoto.mnhn.fr/media/1441450681502sgrit1pvEf02J2vt	Isolectotype	*Allaeanthus greveanus*
*Ampalis greveanus* Baill.	*Grevé 254* (P-00108326)	http://mediaphoto.mnhn.fr/media/1441450681521fShKblWGJWQPwuDN	Isolectotype	*Allaeanthus greveanus*
*Broussonetia kurzii*	*Griffith (Kew Distrib. 4657)* (K-000357622)	http://apps.kew.org/herbcat/getImage.do?imageBarcode=K000357622	Lectotype	*Allaeanthus kurzii*
*Broussonetia luzonica*	*F. C. Gates & F.Q. Otanes 6663* (Merrill: Species Blancoanae No. 468) (US-00688524)	http://n2t.net/ark:/65665/3ec2ec650-7e9f-4de7-be08-aad13028d806	Neotype	*Allaeanthus luzonicus*
*Allaeanthus glaber*	*O. Warburg 12133* (B-10_0294369)	http://plants.jstor.org/stable/pdf/10.5555/al.ap.specimen.b_10_0294369	Holotype	*Allaeanthus luzonicus*
*Allaeanthus glaber*	*O. Warburg 12133* (NY-00025190)	http://plants.jstor.org/stable/pdf/10.5555/al.ap.specimen.ny00025190	Isotype	*Allaeanthus luzonicus*
*Allaeanthus zeylanicus*	*Thwaites*—*C.P. 2215* (B-10_0294368)	http://plants.jstor.org/stable/pdf/10.5555/al.ap.specimen.b_10_0294368	Isotype	*Allaeanthus zeylanicus*
*Allaeanthus zeylanicus*	*Thwaites*—*C.P. 2215* (FR-0031966)	http://plants.jstor.org/stable/pdf/10.5555/al.ap.specimen.fr0031966	Isotype	*Allaeanthus zeylanicus*
*Allaeanthus zeylanicus*	*Thwaites*—*C.P. 2215* (GH-00034340)	http://plants.jstor.org/stable/pdf/10.5555/al.ap.specimen.gh00034340	Isotype	*Allaeanthus zeylanicus*
*Allaeanthus zeylanicus*	*Thwaites*—*C.P. 2215* (K-001050115)	http://plants.jstor.org/stable/pdf/10.5555/al.ap.specimen.k001050115	Isotype	*Allaeanthus zeylanicus*
*Allaeanthus zeylanicus*	*Thwaites*—*C.P. 2215* (K-001050116)	http://plants.jstor.org/stable/pdf/10.5555/al.ap.specimen.k001050116	Isotype	*Allaeanthus zeylanicus*
*Allaeanthus zeylanicus*	*Thwaites*—*C.P. 2215* (L-1583394)	http://data.biodiversitydata.nl/naturalis/specimen/L.1583394	Isotype	*Allaeanthus zeylanicus*
*Allaeanthus zeylanicus*	*Thwaites*—*C.P. 2215* (MPU-017376)	http://plants.jstor.org/stable/pdf/10.5555/al.ap.specimen.mpu017376	Isotype	*Allaeanthus zeylanicus*
*Broussonetia rupicola*	*F. T. Wang 10884* (PE-00760682)	http://www.cvh.org.cn/spm/PE/00760682	Holotype	*Broussonetia monoica*
*Smithiodendron artocarpioideum*	*H.T. Tsai 53462* (PE-00025031)	http://www.cvh.org.cn/spm/PE/00025031	Holotype	*Broussonetia papyrifera*
*Smithiodendron artocarpioideum*	*H.T. Tsai 53462* (P06885709)	http://plants.jstor.org/stable/10.5555/al.ap.specimen.p06885709	Isotype	*Broussonetia papyrifera*
*Smithiodendron artocarpioideum*	*H.T. Tsai 53462* (PE-00023979)	http://www.cvh.org.cn/spm/PE/00023979	Isotype	*Broussonetia papyrifera*
*Smithiodendron artocarpioideum*	*H.T. Tsai 53462* (PE-1991398)	http://www.cvh.org.cn/spm/PE/00934142	Isotype	*Broussonetia papyrifera*

### Molecular phylogenetic analyses

To test the monophyly of *Broussonetia* sensu Corner (1962), Clement and Weiblen (2009)’s aligned DNA matrix of chloroplast *ndhF* and nuclear 26S (TreeBASE Study ID S2229) assembled for phylogenetic analyses of Moraceae was adopted, with morphological characters of the matrix excluded. The analyses of Clement and Weiblen (2009) sampled 76 species representing 32 Moraceae genera and *B. papyrifera* was shown as a sister taxon of *Malaisia scandens* (Lour.) Planch. in the tribe Dorstenieae. All three species of sect. *Broussonetia*, plus *B.* ×*kazinoki*, and three of the four species of sect. *Allaeanthus* were sampled (Additional file 1) for phylogenetic analyses. Conditions for PCR amplification of *ndhF* and 26S detailed in Clement and Weiblen (2009) were followed. Phylogenetic analyses were performed using MrBayes v3.2.6 (Ronquist et al. 2012) for Bayesian inferences (BI) and GARLIC (Bazinet et al. 2014) for maximum likelihood (ML) analyses. Based on Akaike Information Criterion implemented in jModeltest 2 (Darriba et al. 2012), the models GTR + I+Γ and TVM + Γ, which were chosen in previous study (Zerega et al. 2005), were selected for 26S and *ndhF*, respectively. For both BI and ML analyses, the matrix was partitioned. For ML analysis, five independent searches and 500 replicates of bootstraps were performed and results were summarized by PAUP v. 4.0a150 (Swofford 2002). For Bayesian inferences, all parameters were unlinked and estimated independently for each data partition. Two analyses were performed in parallel, each with 4 chains of 20 million generations with temperature set to 0.1, and posterior distribution was sampled every 500 generations. Model parameters and tree statistics were summarized in MrBayes and posterior probabilities higher than 0.75 were mapped to the maximum likelihood best tree manually.

## Results and discussion

### Type specimens of *Broussonetia kaempferi* var. *australis*

In the protologue of *Broussonetia kaempferi* var. *australis*, Suzuki (1934) designated his own (“ST”) collection No. 8336 as the type (holotype), stating “*[Typus] ST 8336*—*in silvis secundariis ad Heikôkô prope Sinten (S* *uzuki*-*Tokio Apr. 2, 1933) in Herb. Univ. Imper. Taihoku.*” Currently in the Herbarium of National Taiwan University (TAI), successor of the Herbarium of the Taihoku Imperial University, no collection bearing *T. Suzuki 8336* was located. However, a collection of *T. Suzuki 8362* bearing the stamp of “*Typus*” is labeled as the holotype of *B. kaempferi* var. *australis* T. Suzuki (http://tai2.ntu.edu.tw/Specimen/specimen.php?taiid=118781). Except for the number, all information on the label of *ST 8362*, “*In silvis secundariis ad Heikôkô prope Sinten, Taihoku*-*syû, Taiwan. Suzuki*-*Tokio; 1933.4.2.*”, matches exactly to the protologue. Unfortunately, *ST 8362* is a badly damaged collection, leaving only a branch and a small leaf without diagnosable characters. Following the description of the taxon, Suzuki (1934) wrote “*[Materiae] Typus*-*flor. mas. et fem. ST* ^*(1)*^ *8337 et ST 4629*–*fl. fem.; ST 6841 et ST 8952*-*fruc.; SS* ^*(2)*^ *3484 et ST 10829*-*steril. Fol. non partitis; SS 6042, SS 5998, ST 10827*-*steril. fol. partitis*.” All the materials cited in “*Materiae*” in Suzuki (1934) are thus paratypes and all but two specimens (*ST 8337* and *ST 10829*) are still available in TAI (Table 2). However, after careful examination of these paratypes, all of them should be identified as *B. monoica* sensu Ohba and Akiyama (2014).

### Vouchers of *Broussonetia kaempferi* and *B. kazinoki* cited in Liao (1989, 1991, 1996)

In the treatments of *Broussonetia*, Liao (1989, 1991, 1996) cited three collections of *B. kaempferi* (*Tanaka & Shimada 13557*, *Yamamoto 37610*, and *Onizuka 22022*) and two collections of *B. kazinoki* (*Liao & Wang 12332* and *Liao 211714*). For *B. kaempferi*, two collections of *Tanaka & Shimada 13557* deposited in PH (Chung et al. 2009) and *Yamamoto 37610* at TAI are available online (Table 2). For *B. kazinoki*, *Liao 211714* was located in TAI. However, despite their determination by Liao (1989, 1991, 1996), all the voucher specimens cited should be identified as *B. monoica* sensu Ohba and Akiyama (2014).

### Identity of *Broussonetia kaempferi* var. *australis*

**Fig. 1:**
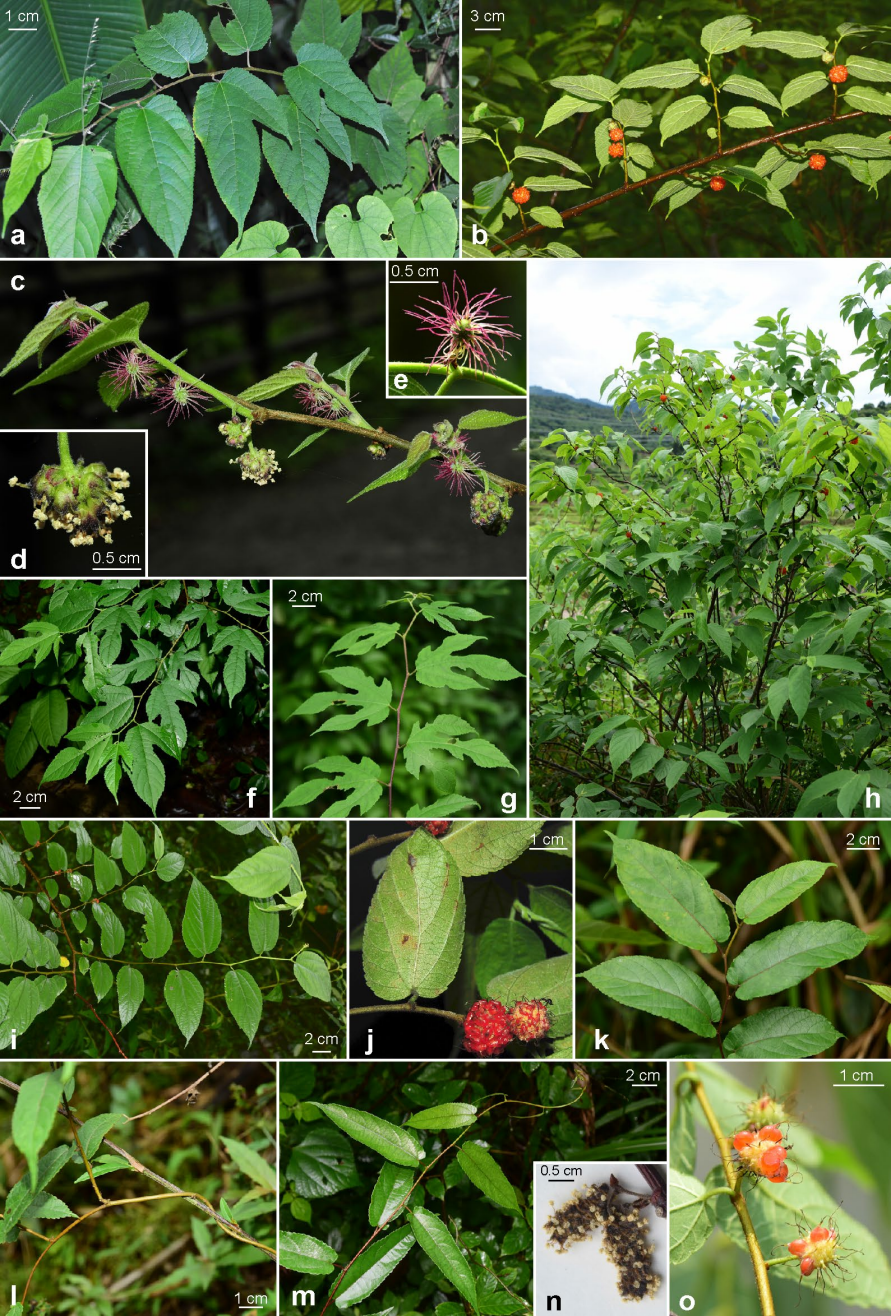
*Broussonetia monoica* Hance (**a**–**j**) and *B. kaempferi* Siebold (**k**–**o**). **a**, **f**, **g**, **i** Variation in leaf morphology; **b** fruiting branch; **c** flowering branch, showing staminate catkins (**d**) and pistillate capitula (**e**); **h** habit; **j** leaves and syncarps; **k** leaves; **l**, **m** habit of *B. kaempferi*, a spiralingly twining liana; **n** spicate staminate catkins; **o** syncarps. [**a** Shiding, New Taipei City, Taiwan, 7 April 2016, *Chung 3332* (HAST); **b** Xianju, Zhejiang, China, 29 May 2016, *Chung 3384* (HAST); **c**–**e** Wulai, New Taipei City, Taiwan, 16 March 2014, *Chung 3335*; **f**, **g** Pujiang, Zhejian, China, 27 May 2016, *Chung 3364* (HAST); **h** Xianju, Zhejiang, China, 28 May 2016, *Chung 3383* (HAST); **i** Xianju, Zhejiang, China, 29 May 2016, *Chung 3384* (HAST); **j** Shiding, New Taipei City, Taiwan, 17 May 2014; **k**–**m**, **o** Zong County, Guangxi, China, 18 April 2016, *Peng 24753*; **n** Yizhang, Hunan, China, 10 March 2004, *Xiao 3316* [E])]

Over the past few years, we have observed several wild populations in Taiwan that matched to the protologue and paratypes of *B. kaempferi* var. *australis* described in Suzuki (1934). Figure 1 summaries their morphological variation and key characteristics. Together with observations of herbarium specimens at A, BM, E, GH, HAST, K, TAI, TAIF, and TNM, we conclude that all Taiwanese materials are monoecious with globose staminate catkins (Fig. 1c–e), the key characteristics of *B. monoica* sensu Ohba and Akiyama (2014). We did not find any living or herbarium collections of Taiwan bearing spicate staminate catkins (Fig. 1n) that are characteristic of *B. kaempferi* (Ohba and Akiyama 2014).

### Molecular phylogenetic analyses

**Fig. 2:**
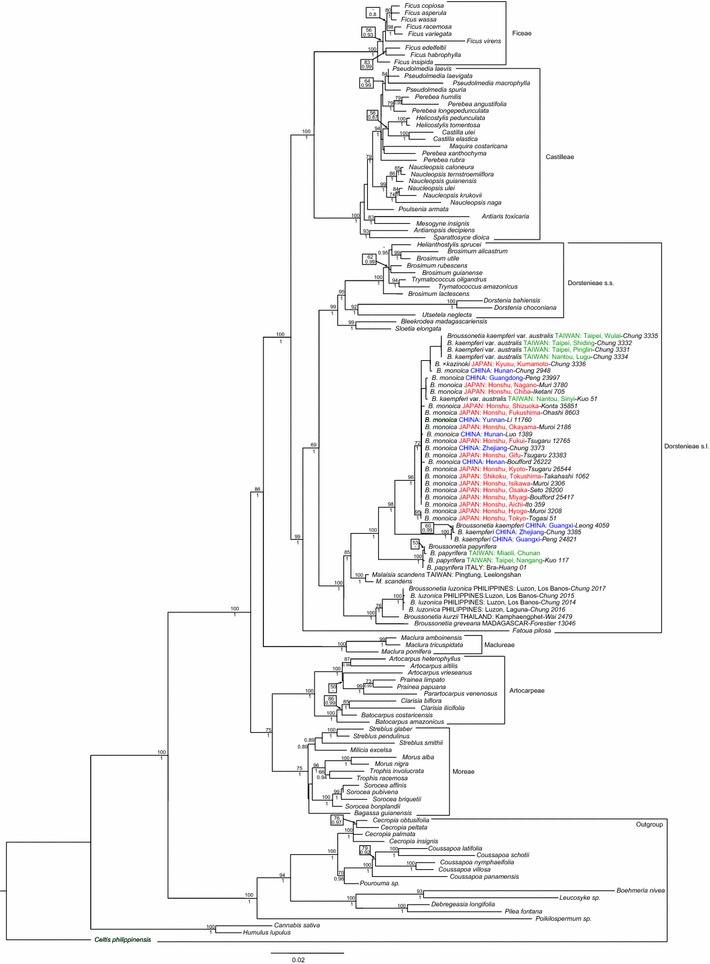
Maximum likelihood tree based on chloroplast *ndhF* and nuclear 26S sequences. Bootstrap percentage ≥50 are labeled above branches. Bayesian posterior probability values ≥0.75 are labeled under branches. Linages obtained in this study are followed by collection sites (Country: locality), collectors and original collection numbers. All Taiwanese samples of *Broussonetia monoica* (*collection sites in green*) would be identified as *B. kaempferi* var. *australis* sensu Suzuki (1934)

Topologies of BI and ML analyses were identical with differences in support values. Figure 2 depicts results of ML analysis marked with both BI and ML support values. With the additional samples of *Broussonetia* sensu Corner (1962), the overall phylogenetic relationships of current analyses are congruent with Clement and Weiblen (2009), with samples of *Broussonetia* sensu Corner (1962) placed in tribe Dorstenieae (Fig. 2). However, although the monophyly of *Broussonetia* sect. *Allaeanthus* and sect. *Broussonetia* were each strongly supported, *Malaisia scandens* was placed as the sister clade to sect. *Broussonetia*, rendering *Broussonetia* sensu Corner (1962) paraphyletic. To correct the paraphyly of *Broussonetia* sensu Corner (1962), we propose to reinstate the generic status of *Allaeanthus* Thwaites. Alternatively, an expanded *Broussonetia* by including *M. scandens* would not only necessitate further nomenclatural changes but also generate a genus with no obvious diagnostic character.

Within the clade sect. *Broussonetia*, all samples of Taiwan that would be identified as *B. kaempferi* var. *australis* sensu Suzuki (1934), plus the natural hybrid *B.* ×*kazinoki*, were placed in a strongly supported clade of *B. monoica* (Fig. 2), supporting our observations that all Taiwanese materials are part of the highly polymorphic *B. monoica*. All three samples of *B. kaempferi* formed a strongly supported clade sister to the strongly supported clade of *B. monoica*, with the clade of *B. papyrifera* further sister to the clade composed of *B. kaempferi* and *B. monoica*.

Within the clade sect. *Allaeanthus*, *B. kurzii* and *B. greveana* were successively sister to the clade of *B. luzonica* with strongest supports. Although our sampling did not include *Broussonetia zeylanica* (≡*Allaeanthus zeylanicus*), the type species of *Allaeanthus*, we are confident that our analysis will sustain as morphologically *B. luzonica* and *B. zeylanica* are quite similar (Corner 1962), differing from each other merely by the length of staminate catkins (10–26 cm in *B. luzonica* vs. ca. 6 cm in *B. zeylanica*) and margins of leaves (entire vs. serrate) and stipules (entire vs. denticulate).

## Conclusions

### Taxonomic treatment

Our phylogenetic analyses revealed that species of *Broussonetia* sensu Corner (1962) were placed in two clades corresponding to sect. *Allaeanthus* and sect. *Broussonetia*, with *Malaisia scandens* placed sister to the clade of sect. *Broussonetia* with strongest supports. To correct the paraphyly of *Broussonetia* sensu Corner (1962), we propose to reinstate the generic status of *Allaeanthus* Thwaites. Within *Broussonetia* sect. *Broussonetia*, *B. kaempferi* var. *australis* is synonymized under *B. monoica*. The species *B. kaempferi* is not distributed in Taiwan.

***Allaeanthus*** Thwaites, Hooker’s J. Bot. Kew Gard. Misc. 6: 302. 1854.—TYPE: *Allaeanthus zeylanica*

***Allaeanthus zeylanicus*** Thwaites, Hooker’s J. Bot. Kew Gard. Misc. 6: 303, *pl. IX.*-*B*. 1854.—Type: SRI LANKA. Central Province. July 1833, *Thwaites*—*C.P. 2215* (holotype: PDA; isotypes: B [B 10 0294368 image!], FR [FR-0031966 image!], GH [GH00034340 image!], K [K001050115 image!], K [K001050116 image!], L [L. 1583394 image!], MPU [MPU017376 image!]).—*Broussonetia zeylanica* (Thwaites) Corner, Gard. Bull. Singapore 19: 235. 1962.

Distribution. Sri Lanka.

***Allaeanthus luzonicus*** (Blanco) Fern.-Vill. in Fl. Filip. (ed. 3) 4(13A): 198. 1880; Merrill, Sp. Blancoan. 122. 1918.—Neotype (designated by Merrill 1918, p. 122): PHILIPPINES: Luzon, Laguna Province, Los Baños, 14 March 1914, *F.C. Gates & F.Q. Otanes 6663* (Merrill: Species Blancoanae No. 468) (US [00688524 image!]).—*Morus luzonica* Blanco, Fl. Filip. 703. 1837.—*Broussonetia luzonica* (Blanco) Bureau in de Candolle, Prodr. 17: 224. 1873; Merrill, Rev. Blancos Fl. Filip. 78. 1905; Corner, Gard. Bull. Singapore 19: 235. 1962; Berg et al., Fl. Malesiana, Ser. I 17(Part 1): 30, *fig.* *3*. 2006.

*Allaeanthus glaber* Warb. in Perkins, Frag. Fl. Philipp. 3: 166. 1904.—Type: PHILIPPINES. Luzon Isl., Prov. Cagayan, Enrile, *O. Warburg 12133* (holotype: B [B 10 0294369 image!]; isotype: NY [00025190 image!]).—*Allaeanthus luzonicus* var. *glaber* (Warb.) Merr., Enum. Philipp. Fl. Pl. 2: 37. 1923.—*Broussonetia luzonica* var. *glabra* (Warb.) Corner, Gard. Bull. Singapore 19: 235. 1962.

Distribution. Philippines and Indonesia (Sulawesi).

Notes: Type materials of most Blanco’s names, including *Morus luzonica* Blanco, are not known (Merrill 1918; Nicolson and Arculus 2001). Following Nicolson and Arculus (2001), *No 468* of the “illustrative specimen” cited in Merrill (1918)’s *Species Blancoanae* is here taken as the effective neotypification for *Morus luzonica* Blanco.

***Allaeanthus kurzii*** Hook. f, Fl. Brit. India 5(15): 490–491. 1888.**—**Lectotype (designated by Upadhyay et al. 2010, p. 22): MYANMAR (“BURMA”): Herbarium of the late East India Company, Birma, s.d., *Griffith* (Kew Distrib. 4657) [female plant] (K [K000357622 image!]).—*Broussonetia kurzii* (Hook. f.) Corner, Gard. Bull. Singapore 19: 234. 1962; Zhou & Gilbert, Fl. China 5: 27. 2003; Berg et al., Fl. Malesiana, Ser. I 17(Part 1): 30. 2006.

Distribution. China (Yunnan), Vietnam, Laos, Thailand, Myanmar, Bhutan, and India (Assam and Sikkim).

***Allaeanthus greveanus*** (Baill.) Capuron, Fiches Bot. Ess. Forest. Madagascar: Fiche 1. 1968; Adansonia, n.s. 12(3): 386. 1972.—*Ampalis greveanus* Baill. in Grandidier, Hist. Phys. Madagascar *t. 293*-*A*. 1891.—**Lectotype (here designated):** MADAGASCAR. Bekopaka, near Morodava, *H. Grevé 254* (P [P00108324 image!]; isolectotypes: P [P00108325 image!], P [P00108326 image!]).—*Chlorophora greveana* (Baillon) Léandri, Mém. Inst. Sci. Madagascar, Sér. B, Biol. Vég. 1: 18. 1948.—*Maclura greveana* (Baillon) Corner, Gard. Bull. Singapore 19: 237. 1962.—*Broussonetia greveana* (Baillon) C.C.Berg, Bull. Jard. Bot. Belg. 47: 356, *fig.* *21*. 1977.

Distribution. Madagascar.

Notes: Of the three collections of *Grevé 254* at P, P00108324 is here designated as the lectotype because the label of this collection contains the most information.

***Broussonetia*** L’Hér. ex Vent., Tabl. Régn. Vég. 3: 547. 1799, *nom. cons.*—TYPE: *Broussonetia papyrifera* L’Hér. ex Vent.

*Papyrius* Lam., Tabl. Encycl. 4(2): *pl. 762*. 1797, nom. illeg.

*Smithiodendron* H.H. Hu, Sunyatsenia 3(2–3): 106. 1936.

***Broussonetia papyrifera*** (L.) L’Hér. ex Vent., Tabl. Régn. Vég. 3: 547. 1799.—*Morus papyrifera* L., Sp. Pl. 2: 986. 1753.—Lectotype (designated by Florence 1997, p. 146): [icon] ‘*Morus papyrifera*’ in Kaempfer, Amoen. Exot. Fasc., 471, *t.472*. 1712.

*Smithiodendron artocarpioideum* H.H. Hu, Sunyatsenia 3(2–3): 107–109, *pl. 6*. 1936.—Type. CHINA: Yunnan, Shih-pin Hsien, 29 May 1933, *H.T. Tsai 53462* (holotype: PE [1640641 image!]; isotypes: P [P06885709 image!], PE [00025034 image!], PE [00023979 image!], PE [00934142 image!]).

Distribution. The reported distributions of *Broussonetia papyrifera* are highly inconsistent across literature (Table 1), confounded by ancient and recent translocations of the species for multiple purposes around the world (Matthews 1996; Barker 2002; Seelenfreund et al. 2010; Chang et al. 2015). The distribution map in Matthews (1996) includes Japan, Korea, China (northern, central, and southern China), Taiwan, Vietnam, Laos, Thailand, Cambodia, Myanmar, India (Sikkim), island Southeast Asia (excluding the Philippines and Borneo), Melanesia, and Polynesia islands. Chang et al. (2015) showed a high chloroplast haplotype diversity in China, Taiwan, and Indochina, suggesting that these regions are likely native range of the species. Zhou and Gilbert (2003) provided a provincial distribution in China (Anhui, Fujian, Gansu, Guangdong, Guangxi, Guizhou, Hainan, Hebei, Hubei, Hunan, Jiangsu, Jiangxi, Shaanxi, Shandgon, Shanxi, Sichuan, SE Xizang, Yunnan, Zhejiang). In Northeast Asia, the non-native status of *B. papyrifera* in Japan has been repeatedly reported (Ohwi 1965; Kitamura and Murata 1980; Okamoto 2006) while this species is regarded as native in Korea (Yun and Kim 2009). Historically, the fibrous *B. papyrifera* had been introduced to Remote Oceanic islands via SE Asian islands (Matthews 1996; Chang et al. 2015); however, its growth and populations in these regions had declined significantly since last century (Matthews 1996). On the other hand, *B. papyrifera* has been introduced and become naturalized and invasive around the world (Florece and Coladilla 2006; Bosu et al. 2013; Rashid et al. 2014; Chang et al. 2015).

***Broussonetia kaempferi*** Siebold, Verh. Batav. Genootsch. Kunst. 12: 28. 1830; Akiyama et al., J. Jap. Bot. 88: 351. 2013; Ohba & Akiyama, J. Jap. Bot. 89: 127. 2014.—Lectotype (designated by Akiyama et al. 2013, p. 351): [icon] ‘*Papyrus spuria*’ in Kaempfer, Amoen. Exot. Fasc, *t.472*, 474. 1712.

*Broussonetia kaempferi* var. *australis* auct. non T. Suzuki: Yamazaki, J. Phytogeogr. Taxon. 30(2): 69. 1982; Chang et al., Fl. Reipubl. Popul. Sin. 23(1): 27, *pl. 7(9*–*13)*. 1998; Zhou & Gilbert, Fl. China 5: 27. 2003.

Distribution. Japan (Shikoku and Kyushu), central to southern China (Anhui, Chongqing, Fujian, Guangdong, Guangxi, Guizhou, Hubei, Hunan, Jiangxi, Yunnan, and Zhejiang), northern Vietnam, and India (Arunachal Pradesh; Naithani 1981).

Notes. *Broussonetia kaempferi* is not distributed in Taiwan; *B. kaempferi* var. *australis* is a synonym of *B. monoica*. The images of *Broussonetia* ‘*kazinoki*’ in Utteridge and Bramley (2015, p. 77, figs. 2 & 6) are a pistillate individual of *B. kaempferi*.

***Broussonetia*** **×** ***kazinoki*** Siebold (in Verh. Batav. Genootsch. Kunst. 12: 28. 1830, *nom. nud.*) in Siebold & Zuccarini, Abh. Math.-Phys. Cl. Königl. Bayer. Akad. Wiss. 4(3): 221. 1846; Akiyama et al., J. Jap. Bot. 88: 352, *fig.* *44*. 2013; Ohba & Akiyama, J. Jap. Bot. 89: 127. 2014.—Lectotype (designated by Akiyama et al. 2013, p. 352): JAPAN. *von Siebold s.n.* 1842 (M [M-0120984 image!]).

*Broussonetia* ×*hanjiana* M. Kim in Yun and Kim, Korea J. Pl. Taxon. 39: 82. 2009: 82, **syn. nov.** Type: —KOREA. Province Jeonnam, Is. Gageo, 16 May 2008, *M. Kim 9944* (holotype: JNU).

Distribution. Documented from Japan (Kitamura and Murata 1980; Okamoto 2006; Ohba and Akiyama 2014) and Korea (Yun and Kim 2009).

Distribution. Japan and Korea.

Notes. Long regarded as *Broussonetia kazinoki* × *B. papyrifera* (Okamoto 2006), the Japanese Kôzo *Broussonetia* **×** *kazinoki* is actually the natural hybrid between *B. monoica* and *B. papyrifera* cultivated for paper making since ancient time in Japan and Korea (Yun and Kim 2009; Ohba and Akiyama 2014). *Broussonetia* × *kazinoki* is highly variable and “*various intermediate forms are known between the parent species* (i.e., *B. monoica* and *B. papyrifera*) *in such features as plant sex (dioecious or monoecious), hairness of young shoots, and leaf shape and texture*” (Okamoto 2006). Yun and Kim (2009) reports that *B.* **×** *hanjiana* (≡ *B.* **×** *kazinoki*) is dioecious. Further study is needed to understand the origins of this natural hybrid.

***Broussonetia monoica*** Hance, J. Bot. 20 (238): 294. 1882; Ohba & Akiyama, J. Jap. Bot. 89: 127. 2014.—Type: CHINA. Guangdong (“*prov. Cantonensis*”), “*Lien chau*”, 1881, *B. C. Henry 21933* (holotype: BM [BM000895739 image!]).

*Broussonetia kaempferi* auct. non Siebold: Hayata, J. Coll. Sci. Imp. Univ. Tokyo. 30: 273. 1911; Kanehira, Formos. Trees rev. ed. 146. 1936; Li, Woody Flora of Taiwan 113, *fig.* *35*. 1963; Liao, Quart. J. Exp. Forest. 3(1): 148. 1989; Liu et al., Trees of Taiwan 331. 1994, *pro parte*; Liao, Fl. Taiwan, 2nd. ed. 2: 140. 1996, *pro parte*; Lu et al., Trees of Taiwan 2: 95, photos. 2006, *pro parte*.

*Broussonetia kaempferi* var. *australis* T.Suzuki, Trans. Nat. Hist. Soc. Taiwan 24: 433–435. 1934.—Type: TAIWAN. “*In silvs secundris ad Heikoko prope Sinten*”, *T. Suzuki 8362* (“*ST 8336*”), 2 Apr 1933 (holotype: TAI [118781 image!]).

*Broussonetia rupicola* F.T. Wang & Tang, Acta Phytotax. Sin. 1(1): 128. 1951.—Type: CHINA. “Szechuan” (Sichuan), Nanchuan, *F. T. Wang 10884* (holotype: PE [00760682 image!]), **syn. nov.**

*Broussonetia jiangxiensis* X.W Yu, J. Jiangxi Agric. Univ. (1): 3, *fig. 2*. 1982—Type: CHINA. Jiangxi, Nanchang, *X.W Yu 1435* (holotype: JXAU), **syn. nov.**

*Broussonetia kazinoki* var. *ruyangensis* P.H.Liang & X.W.Wei, Bull. Bot. Res., Harbin 2(1): 155–156, *fig. 1.* 1982.—Type: CHINA. Guangdong: Ruyang, Wu-Zhi-Shan, 600–800 m, 28 Mar 1979, *X.*-*W. Wei 4471* (holotype: CANT).

*Broussonetia kazinoki* form. *koreana* M. Kim, Korean J. Pl. Taxon. 39(2): 84, *fig. 1F, 1G*. 2009.—Type: KOREA. Province Jeonnam, Is. Gageo, 16 May 2008, *M. Kim 9946* (holotype: JNU), **syn. nov.**

*Broussonetia kazinoki* auct. non Siebold: Liu, Illustrations of Native and Introduced Ligneous Plants of Taiwan 2: 707, *pl. 561*. 1962; Liu & Liao, Fl. Taiwan 2: 120, 122, *pl. 234*. 1976; Liao, Quart. J. Exp. Forest. 3(1): 148–149. 1989; Liu et al., Trees of Taiwan 331. 1994, *pro parte*; Liao, Fl. Taiwan, 2nd. ed. 2: 140, *pl. 68*, *photo 59*. 1996, *pro parte*; Chang et al., Fl. Reipubl. Popul. Sin. 23(1): 26, *pl. 7(6*–*8)*, 1998; Zhou & Gilbert, Fl. China 5: 26–27. 2003; Lu et al., Trees of Taiwan 2: 95, *photos*. 2006, *pro parte*; Yun & Kim, Korean J. Pl. Taxon. 39(2): 84, *fig. 1C, 1F, 1G*. 2009.

Distribution. Japan (Honshu, Kyushu, Shikoku), Korea, central to southern China (Anhui, Fujian, Guangdong, Guangxi, Guizhou, Hainan, Henan, Hubei, Hunan, Jiangsu, Jiangxi, Sichuan, Yunnan, Zhejiang), Taiwan, and northern Vietnam.

Notes. Until the lectotypification of Siebold’s Japanese collections (Akiyama et al. 2013) and subsequent taxonomic revision of Japanese *Broussonetia* (Ohba and Akiyama 2014), this monoecious *Broussonetia* had long been mis-treated as *B. kazinoki*, which should be applied to the natural hybrid between *B. monoica* and *B. papyrifera*.

Leaves of *B. monoica* are highly polymorphic, varying considerably even within an individual throughout the growing season (Fig. 1). Specimens of *B. monoica* bearing undivided obovate to lanceolate leaves (Fig. 1i, j) are extremely similar to and difficult to be distinguished from *B. kaempferi*; misidentification and confusion of the two species are common both in herbarium collections and the literature. The most important and unambiguous diagnostic character that separates the two species is the shape of staminate catkins, with the dioecious *B. kaempferi* bearing spicate catkins ca. 1.5–2.5 cm long (Fig. 1n) and the monoecious *B. monoica* bearing globose ones ca. 1 cm across (Fig. 1c, d). However, based on our field observation, the globose staminate catkins of *B. monoica* flowers are extremely fragile and caducous during its flowering season in early spring, falling off shortly after their appearance. Consequently, it is highly probable that individuals bearing only the pistillate capitula are misidentified as female plants of *B. kaempferi*. Under this circumstance, sterile individuals of the two species can be distinguished by their growth habit and leaf morphology. *Broussonetia kaempferi* is a climbing and often twining liana (Fig. 1l, m) whereas *B. monoica* is a shrub often with slender twigs (Fig. 1f–h). Leaves of *B. kaempferi* are thinly chartaceous, narrowly oblong to lanceolate with almost symmetric (sub-)cordate leaf base and undivided and crenate margin. In contrast, leaves of *B. monoica* are thinly herbaceous and highly variable, ranging from oblique ovate or broadly ovate (Fig. 1a, b, f, g) similar to *Morus australis* Poir. (e.g., Pl. 68 Liao 1996) to narrowly ovate (Fig. 1i, j) similar to *B. kaempferi*.

## Additional file

**Additional file 1.** ***Taxon***, voucher information [*collector No.* (herbarium acronym), Country of origins (Locality)], and NCBI accession numbers (26S/*ndhF*) of newly collected DNA sequences.
