# Supplementary material for: Molecular recircumscription of Broussonetia (Moraceae) and the identity and taxonomic status of B. kaempferi var. australis
Source: Bot Stud. 2017 Feb 21;58:11. doi: 10.1186/s40529-017-0165-y (PMC5432938; doi:10.1186/s40529-017-0165-y)
Supplement: Supplementary file 1 — Additional file 1. Taxon, voucher information [collector No. (herbarium acronym), Country of origins (Locality)], and NCBI accession numbers (26S/ndhF) of newly collected DNA sequences. [file 40529_2017_165_MOESM1_ESM.docx]

**Additional File: *Taxon***, voucher information [*collector* *No.* (herbarium acronym), Country of origins (Locality)], and NCBI accession numbers (26S/*ndhF*) of newly collected DNA sequences.

***Broussonetia greveana***, *Service Forestier Madagascar 13046* (A), Madagascar (Tulear), KY088132/KY088144; ***Broussonetia kaempferi***, *Chung 3385* (HAST), China (Zhejiang, Lishui), KY088122/KY088145; ***Broussonetia kaempferi***, *Leong 4059* (HAST), China (Guangxi, Huanjiang), KY088123/KY088146; ***Broussonetia kaempferi***, *Peng 24821* (HAST), China (Guangxi, Longzhou), KY088124/KY088147; ***Broussonetia* ×*kazinoki***, *Chung 3336* (HAST), Japan (Kumamoto), KY088120/KY088158; ***Broussonetia kurzii***, *Wai 2479* (HAST), Thailand (Kamphaengphet), KY088133/KY088148; ***Broussonetia luzonica***, *Chung 2014*, Philippines (Luzon, Los Baños), KY088096/KY088149; ***Broussonetia luzonica***, Chung 2015 (HAST), Philippines (Luzon, Los Baños), KY088097/KY088150; ***Broussonetia luzonica***, *Chung 2016* (HAST), Philippines (Luzon, Laguna), KY088098/KY088151; ***Broussonetia luzonica***, *Chung 2017* (HAST), Philippines (Luzon, Los Baños), KY088138/KY088152; ***Broussonetia monoica***, *Chung 2948* (HAST), China (Hunan, Dao County), KY088099/KY088153; ***Broussonetia kaempferi* var. *australis***, *Chung 3331* (HAST), Taiwan (New Taipei City, Pinglin), KY088100/KY088154; ***Broussonetia kaempferi* var. *australis***, *Chung 3332* (HAST), Taiwan (New Taipei City, Shiding), KY088104/KY088155; ***Broussonetia kaempferi* var. *australis***, *Chung 3334* (HAST), Taiwan (Nantou, Lugu), KY088102/KY088156; ***Broussonetia kaempferi* var. *australis***, *Chung 3335* (HAST), Taiwan (New Taipei City, Wulai), KY088103/KY088157; ***Broussonetia kaempferi* var. *australis***, *Chung 33 73* (HAST), China (Zhejiang, Lishui), KY088105/KY088159; ***Broussonetia kaempferi* var. *australis***, *Kuo 51* (HAST), Taiwan (Nantou, Sinyi), KY088108/KY088178; ***Broussonetia monoica***, *Peng 23997* (HAST), China (Guangdong, Qingyuan), KY088106/KY088179; ***Broussonetia monoica***, *Ito 359* (A), Japan (Aichi), KY088111/KY088160; ***Broussonetia monoica***, *Iketani 705* (A), Japan (Chiba), KY088139/KY088161; ***Broussonetia monoica***, *Tsugaru 12765* (A), Japan (Fukui), KY088116/KY088162; ***Broussonetia monoica***, *Ohashi 8603* (A), Japan (Fukushima), KY088140/KY088163; ***Broussonetia monoica***, *Tsugaru 23383* (A), Japan (Gifu), KY088112/KY088164; ***Broussonetia monoica***, *Muroi 3208* (A), Japan (Hyogo), KY088141/KY088165; ***Broussonetia monoica***, *Muroi 2306* (A), Japan (Isikawa), KY088137/KY088166; ***Broussonetia monoica***, *Tsugaru 26544* (A), Japan (Kyoto), KY088136/KY088167; ***Broussonetia monoica***, *Boufford 25417* (A), Japan (Miyagi), KY088117/KY088168; ***Broussonetia monoica***, *Muroi 3780* (A), Japan (Nagano), KY088118/KY088169; ***Broussonetia monoica***, *Muroi 2186* (A), Japan (Okayama), KY088142/KY088170; ***Broussonetia monoica***, *Seto 28200* (A), Japan (Osaka), KY088110/KY088171; ***Broussonetia monoica***, *Konta 35851* (A), Japan (Shizuoka), KY088109/KY088172; ***Broussonetia monoica***, *Togasi 51* (A), Japan (Tokyo), KY088143/KY088173; ***Broussonetia monoica***, *Takahashi 1062* (A), Japan (Tokushima), KY088107/KY088174; ***Broussonetia monoica***, *Boufford 26222* (A), China (Henan Neixiang), KY088119/KY088175; ***Broussonetia monoica***, *Luo 1389* (A), China (Hunan, Xining), KY088114/KY088176; ***Broussonetia monoica***, *Li 11760* (A), China (Yunnan, Gongshan), KY088115/KY088177; ***Broussonetia papyrifera***, *Kuo 117* (HAST), Taiwan (Taipei, Nangang), KY088125/KY088180; ***Broussonetia papyrifera***, Italy (Bra), KY088130/KY088181; ***Broussonetia papyrifera***, Taiwan (Miaoli, Chunan), KY088131/KY088182; ***Malaisia scandens***, Taiwan (Pingtung, Leelongshan), KY088135/KY088183.
